# Association of Triglyceride to high-density lipoprotein cholesterol ratio and incident of diabetes mellitus: a secondary retrospective analysis based on a Chinese cohort study

**DOI:** 10.1186/s12944-020-01213-x

**Published:** 2020-03-04

**Authors:** Zhuangsen Chen, Haofei Hu, Miaoling Chen, Xueying Luo, Weili Yao, Qian Liang, Fan Yang, Xinyu Wang

**Affiliations:** 1 Department of Endocrinology, The First Affiliated Hospital of Shenzhen University, No.3002 Sungang Road, Futian District, Shenzhen, 518000 Guangdong Province China; 2 Department of Endocrinology, Shenzhen Second People’s Hospital, Shenzhen, 518000 Guangdong Province China; 3 Shenzhen University Health Science Center, Shenzhen, 518000 Guangdong Province China; 4 Department of Nephrology, The First Affiliated Hospital of Shenzhen University, Shenzhen, 518000 Guangdong Province China; 5 Department of Nephrology, Shenzhen Second People’s Hospital, Shenzhen, 518000 Guangdong Province China; 6 Department of Plastic and reconstructive, Shenzhen People’s Hospital, Shenzhen, 518000 Guangdong China; 7 Department of Endocrinology, Shenzhen Longhua District People’s Hospital, Shenzhen, 518000 Guangdong Province China; 8 Department of Endocrinology, Shenzhen People’s Hospital, Shenzhen, 518000 Guangdong Province China

**Keywords:** Triglycerides, High-density lipoprotein cholesterol, Incident diabetes, Nonlinearity

## Abstract

**Background:**

Previous studies have revealed that triglyceride to high-density lipoprotein cholesterol ratio (TG/HDL-C) is one of major risk factors of insulin resistance and diabetes. However, study on the association between TG/HDL-C and diabetes mellitus (DM) risk is limited, especially in Chinese people. This study was undertaken to investigate the relationship between TG/HDL-C and incident of diabetes in a large cohort in Chinese population.

**Methods:**

The present study was a retrospective cohort study. A total of 114,787 adults from Rich Healthcare Group in China, which includes all medical records for participants who received a health check from 2010 to 2016. The target independent variable and the dependent variable were triglyceride to high-density lipoprotein cholesterol ratio measured at baseline and incident of diabetes mellitus appeared during follow-up respectively. Covariates involved in this study included age, gender, body mass index, diastolic blood pressure, systolic blood pressure, fasting plasma glucose, total cholesterol, low density lipoprotein cholesterol, serum creatinine, smoking and drinking status and family history of diabetes. Cox proportional-hazards regression was used to investigate the association of TG/HDL-C and diabetes. Generalized additive models was used to identify non-linear relationships. Additionally, we also performed a subgroup analysis. It was stated that the data had been uploaded to the DATADRYAD website.

**Result:**

After adjusting age, gender, body mass index, systolic blood pressure, diastolic blood pressure, fasting blood glucose, total cholesterol, low density lipoprotein cholesterol, serum creatinine, smoking and drinking status and family history of diabetes, result showed TG/HDL-C was positively associated with incident of diabetes mellitus (HR = 1.159, 95%CI (1.104, 1.215)). A non-linear relationship was detected between TG/HDL-C and incident of diabetes, which had an inflection point of TG/HDL-C was 1.186. The effect sizes and the confidence intervals on the left and right sides of the inflection point were 1.718(1.433,2.060) and 1.049(0.981,1.120), respectively. Subgroup analysis showed, the stronger association can be found in the population with fasting plasma glucose (FPG) < 6.1 mmol/L (P for interaction< 0.0001; HR = 1.296 with FPG < 6.1 mmol/L vs HR = 1.051 with FPG ≥ 6.1 mmol/L).The same trend was also seen in the population with body mass index (BMI)(≥18.5, < 24 kg/m^2^) (P for interaction = 0.010,HR = 1.324) and family history without diabetes(P for interaction = 0.025, HR = 1.170).

**Conclusion:**

TG/HDL-C is positively associated with diabetes risk. The relationship between TG/HDL-C and incident of diabetes is also non-linear. TG/HDL-C was strong positively related to incident of diabetes when TG/HDL-C is less than 1.186.

## Background

Diabetes has become one of the most common chronic diseases all over the world. Diabetes can cause directly or indirectly damage to people and cause metabolic abnormalities and various complications, which is harm to health and survival, resulting in lower quality of life and increased risk of death. The prevalence of diabetes in adults has increased significantly in recent decades in China [1]. According to reports, the prevalence of diabetes in adults is 10.4% in China in 2013 [2]. Therefore, it is important to explore and intervene in the risk factors for diabetes.

Diabetes is often associated with abnormal lipid metabolism, which is an important risk factor for diabetic vascular disease. Dyslipidemia is characterized by elevated blood triglyceride (TG) levels and decreased high-density lipoprotein cholesterol (HDL-C) levels in diabetes and insulin resistance [3]. Some researchers revealed triglyceride to high-density lipoprotein cholesterol ratio(TG/HDL-C) is closely related to insulin resistance [4, 5], and also associated with obesity and metabolic syndrome [6]. Meanwhile, some studied explored the association between TG/HDL-C and diabetes. They showed positive association between TG/HDL-C and diabetes [7–10], while one study reached inconsistent results [11]. In Chinese, two studies [7, 9] found that TG/HDL-C independently increased the Type 2 diabetes (T2DM) risk in a general population, however, the relatively small sample size and regional population limited to generalizable other people. Therefore, this study set out to investigate whether TG/HDL-C was independently related to the risk of incident diabetes in a large cohort population across 32 sites and 11 cities in China.

In this study, we performed a secondary data analysis based on previously published data. In that paper, the author investigated the correlation between body mass index (BMI) and the risk of incident diabetes [12]. On secondary analysis, TG/HDL-C was used as an independent variable, and outcome variables and other covariates are consistent with those in the original analysis.

## Methods

### Data source and participants

Date were obtained from ‘DATADRYAD’ database (www.Datadryad.org). This website permitted users to freely download the raw data. According to Dryad Terms of Service, we cited Dryad data package in the present study. (Dryad data package: Ying Chen, Xiao-Ping Zhang, Jie Yuan, Bo Cai, Xiao-Li Wang, Xiao-Li Wu, Yue-Hua Zhang, Xiao-Yi Zhang, Tong Yin, Xiao-Hui Zhu, Yun-Juan Gu1, Shi-Wei Cui, Zhi-Qiang Lu, Xiao-Ying Li (2018) Data from: Association of body mass index and age with incident diabetes in Chinese adults: a population-based cohort study. Dryad Digital Repository. 10.1136/bmjopen-2018-021768). Variables included in the database file were as follows: age, gender,body mass index (BMI), diastolic blood pressure (DBP), systolic blood pressure (SBP), fasting plasma glucose (FPG), Triglyceride(TG), total cholesterol (TC), low density lipoprotein cholesterol (LDL-C), high density lipoprotein cholesterol (HDL-C), Serum urea nitrogen (BUN), Serum creatinine (Scr), smoking status, drinking status, family history of diabetes, year of follow up and censor of diabetes at follow up. Authors of the original study have waived all copyright and related ownership of these data. Therefore, we could use these data for secondary analysis without infringing on the authors’ rights.

Data were obtained from a database provided by the Rich Healthcare Group in China, and the study enrolled 685,277 participants who received a health check and were at least 20 years old with at least two visits between 2010 and 2016 across 32 sites and 11 cities in China (Shanghai, Beijing, Nanjing, Suzhou, Shenzhen, Changzhou, Chengdu, Guangzhou, Hefei, Wuhan, Nantong). The data we got has been initially screened, as follows:(1) no available information about weight, height, gender, fasting plasma glucose value at baseline, (2) extreme BMI values (< 15 kg/m^2^ or > 55 kg/m^2^), (3) excluded participants with visit intervals less than 2 years, (4) participants diagnosed with diabetes at baseline and participants with undefined diabetes status at follow-up. Finally, Ying Chen, et al. [12] selected 211,833 participants in the analysis. Details regarding inclusion/exclusion criteria and outcome measures of the trial have been described in that retrospective cohort study [12]. The institutional ethics committee did not require any obtainment of study approval or informed consent for the retrospective component of the research. For further research, we were excluded missing values of baseline TG (*n* = 5747) and HDL-C (*n* = 89,231) from the analysis cohort. And then TG/HDL-C was calculated as TG divided by HDL-C, we excluded outliers of TG/HDL-C(<means minus three standard deviation (SD) or > means plus three SD) (*n* = 2068) [13]. The final analysis included 114,787 subjects (61,097 male and 53,690 female) for data analysis in our study.

### Study design and measurement of variables

Researchers obtained information (values) for our retrospective cohort study. The design of the study has been documented elsewhere [12]. In order to allow to understand the entire research process more clearly, we have outlined the steps of the study here. In each visit to the health check centre, participants were requested to complete a detailed questionnaire regarding demographic characteristics, lifestyle factors, personal medical history and family history of chronic disease. Subjects were measured for height, weight and blood pressure by trained staff. Body weight was measured in light clothing with no shoes to the nearest 0.1 kg. Height was measured to the nearest 0.1 cm. BMI was derived from weight in kilograms divided by height in metres squared. Blood pressure was measured by standard mercury sphygmomanometers. Fasting venous blood samples were collected after at least a 10 h fast at each visit. TG, TC, LDL-C and HDL-C were measured on an autoanalyzer (Beckman 5800). Plasma glucose levels were measured by the glucose oxidase method on an autoanalyzer (Beckman 5800). The target independent variable is TG/HDL-C obtained at baseline. The dependent variable is incident diabetes obtained in the follow up. As this is a retrospective cohort study, reducing the possibility of selection bias and observation bias.

### Ascertainment of incident diabetes

Diagnosis of incident diabetes was defined as fasting plasma glucose of > 7.00 mmol/L and/or self-reported diabetes during the follow-up period. Patients were censored at the date of diagnosis of diabetes or the final visit, whichever came first. The number of people lost to follow-up is still included in the study.

### Statistical analysis

First, we handled missing values of other variable.While the missing data was continuous variable, we supplemented with the mean or median. When it was categorical variable, we treated this variable as a categorical variable [14].

Next, the participants were stratified by quartiles of TG/HDL-C. Continuous variables were expressed as the means ± standard deviations (normal distribution) or medians (quartiles) (skewed distribution),and categorical variables were expressed as a frequency or percentages. The one-way ANOVA (normal distribution), Kruskal-Wallis H (skewed distribution) test and chi-square test (categorical variables) were used to determine any significant differences between the means and proportions of the groups. Cox proportional hazard regression models were used to investigate the prognostic value of TG/HDL-C on diabetes risk, and adjusted hazard ratios (HRs) with 95% confidence intervals (CIs) were estimated to evaluate the risk of diabetes. According to the recommendation of the STROBE statement [15], we simultaneously showed the results from unadjusted, minimally adjusted analyses and those from fully adjusted analyses. Whether the covariances were adjusted determined by the following principle: when added to this model, changed the matched hazard ratio by at least 10% [16]. In addition, we also analyzed the association between TG,HDL-C,TG/LDL-C,LDL-C/HDL-C,TC/HDL-C,TC/LDL-C and diabetes risk. To ensure the robustness of data analysis, we did a sensitivity analysis. We converted the TG/HDL-C into a categorical variable, and calculated the P for trend. The purpose was to verify the results of TG/HDL-C as the continuous variable and to observe the possibility of nonlinearity. We also tried to use generalized additive models (GAM) to identify non-linear relationships because TG/HDL-C was a continuous variable. If a non-linear correlation was observed, a two-piecewise linear regression model was performed to calculate the threshold effect of the TG/HDL-C on incident of diabetes in terms of the smoothing plot. When the ratio between incident of diabetes and TG/HDL-C appeared obvious in a smoothed curve, the recursive method automatically calculates the inflection point, where the maximum model likelihood will be used. Robustness of the results in various subgroups(age, gender, BMI, FPG, SBP, DBP, family history of diabetes, smoking and drinking status) was also explored by Cox proportional hazard models. For continuous variable, we first converted it to a categorical variable according to the clinical cut point or binary. Each stratification was adjusted for all the factors, except for the stratification factor itself. The modifications and interactions of subgroups were inspected by likelihood ration tests. Survival estimates and cumulative event rates were compared using the Kaplan–Meier method by using the time-to-first event for each endpoint. The log-rank test was used to compare the Kaplan–Meier hazard ratios (HR) for adverse events, and their corresponding 95% confidence intervals (CIs).

All of the analyses were performed with the statistical software package R (http://www.R-project.org, The R Foundation) and Empower-Stats (http://www.empowerstats.com, X&Y Solutions,Inc., Boston, MA). *P* values less than 0.05 (two-sided) were considered statistically significant.

## Results

A total of 114,787 participants (53.2% men and 46.8% women) were included in the analysis, the mean age of the population was 44.0 ± 12.9 years old. The mean year of follow up was 3.1 ± 0.9 years, and 2512 people developed diabetes during follow-up. The mean TG/HDL-C was 1.0 ± 0.7, and the mean FPG and BMI were 4.9 ± 0.6 mmol/L and 23.3 ± 3.3 kg/m^2^,respectively. The number of participants with missing data of SBP, DBP, Scr and LDL-C were 18,18,1341and 192, respectively. Meanwhile, the missing data of smoking and drinking status were 84,169 and 84,169.

### Baseline characteristics of the study participants

Table 1 depicted the baseline characteristics of the total population and by quartiles of the TG /HDL-C. We assigned participants into subgroup using TG/HDL-C quartiles (≤0.52, 0.52–0.80, 0.80–1.30, > 1.30). We found that in highest TG/HDL-C group, participants generally had higher age, BMI, blood pressure levels (including both systolic and diastolic blood pressures), fasting blood glycemic, TC, LDL-C, Scr and higher rates of current smoker and drinker. In contrast, There was no statistically significant difference in family history of diabetes among different TG/HDL-C groups.

**Table 1:** The Baseline Characteristics of participants

TG/HDL-C	Q1(≤0.52)	Q2(0.52 to ≤0.80)	Q3(0.80 to ≤1.30)	Q4(> 1.30)	***P***-value
Participants	28,687	28,698	28,705	28,697	
AGE(years)	40.11 ± 11.43	43.14 ± 12.92	45.53 ± 13.30	47.25 ± 12.92	< 0.001
Gender					< 0.001
Male	7881 (27.47%)	13,547 (47.21%)	17,820 (62.08%)	21,849 (76.14%)	
Female	20,806 (72.53%)	15,151 (52.79%)	10,885 (37.92%)	6848 (23.86%)	
BMI(kg/m^2^)	21.35 ± 2.61	22.58 ± 2.95	23.94 ± 3.10	25.32 ± 3.03	< 0.001
SBP(mmHg)	113.17 ± 14.93	117.59 ± 16.10	121.39 ± 16.65	124.96 ± 16.43	< 0.001
DBP(mmHg)	70.33 ± 9.89	73.03 ± 10.40	75.51 ± 10.84	78.41 ± 10.90	< 0.001
FPG(mmol/L)	4.80 ± 0.54	4.90 ± 0.57	4.98 ± 0.61	5.08 ± 0.65	< 0.001
TC(mmol/L)	4.55 ± 0.80	4.66 ± 0.85	4.84 ± 0.89	5.07 ± 0.92	< 0.001
LDL-C(mmol/L)	2.56 ± 0.59	2.71 ± 0.64	2.86 ± 0.67	2.96 ± 0.72	< 0.001
Scr(mmol/L)	64.02 ± 13.75	68.95 ± 15.85	72.51 ± 15.72	75.44 ± 15.10	< 0.001
Smoking status					< 0.001
Never smoker	6063 (21.14%)	6242 (21.75%)	6052 (21.08%)	5974 (20.82%)	
Ever smoker	143 (0.50%)	283 (0.99%)	404 (1.41%)	457 (1.59%)	
Current smoker	570 (1.99%)	1142 (3.98%)	1777 (6.19%)	2880 (10.04%)	
Not recorded	21,911 (76.38%)	21,031 (73.28%)	20,472 (71.32%)	19,386 (67.55%)	
Drinking status					< 0.001
Never drinker	5969 (20.81%)	6298 (21.95%)	6466 (22.53%)	7018 (24.46%)	
Ever drinker	719 (2.51%)	1221 (4.25%)	1515 (5.28%)	1938 (6.75%)	
Current drinker	88 (0.31%)	148 (0.52%)	252 (0.88%)	355 (1.24%)	
Not recorded	21,911 (76.38%)	21,031 (73.28%)	20,472 (71.32%)	19,386 (67.55%)	
Family history of diabetes					0.360
NO	28,068 (97.84%)	28,041 (97.71%)	28,072 (97.79%)	28,019 (97.64%)	
YES	619 (2.16%)	657 (2.29%)	633 (2.21%)	678 (2.36%)	

Values are n(%) or mean ± SD

*BMI* Body mass index, *SBP* Systolic blood pressure, *DBP* Diastolic blood pressure, *FPG* Fasting plasma glucose, *TC* Total cholesterol, *LDL-C* Low-density lipid cholesterol, *Scr* Serum creatinine

### Univariate analysis

The results of univariate analysis were shown in Table 2. The results of univariate analysis showed that age, BMI, SBP, DBP, FPG, TC, LDL, family history of diabetes, smoking and drinking status were positively correlated with incident of diabetes. We also found that women have a lower risk of developing diabetes than men.

**Table 2:** The results of univariate analysis

	Statistics	HR (95% CI)	*P* value
Age	44.08 ± 12.93	1.06 (1.06, 1.07)	< 0.0001
Gender			
Male	62,868 (53.80%)	ref	
Female	53,987 (46.20%)	0.50 (0.46, 0.54)	< 0.0001
BMI	23.35 ± 3.30	1.22 (1.21, 1.23)	< 0.0001
FPG	4.95 ± 0.61	9.98 (9.45, 10.54)	< 0.0001
TC	4.79 ± 0.90	1.34 (1.29, 1.39)	< 0.0001
TG/HDL-C	1.02 ± 0.71	1.83 (1.76, 1.90)	< 0.0001
SBP	119.42 ± 16.68	1.04 (1.04, 1.04)	< 0.0001
DBP	74.44 ± 10.97	1.04 (1.04, 1.05)	< 0.0001
LDL-C	2.77 ± 0.68	1.35 (1.28, 1.42)	< 0.0001
Scr	70.34 ± 15.73	1.01 (1.01, 1.01)	< 0.0001
Smoking status			
Never smoker	24,686 (21.13%)	ref	
Ever smoker	1328 (1.14%)	2.04 (1.50, 2.77)	< 0.0001
Current smoker	6672 (5.71%)	2.37 (2.02, 2.77)	< 0.0001
Not recorded	84,169 (72.03%)	1.54 (1.38, 1.71)	< 0.0001
Drinking stauts			
Never drinker	26,273 (22.48%)	ref	
Ever drinker	5535 (4.74%)	0.93 (0.76, 1.14)	0.4999
Current drinker	878 (0.75%)	1.94 (1.35, 2.79)	0.0003
Not recorded	84,169 (72.03%)	1.17 (1.06, 1.28)	0.0013
Family history of diabetes			
No	114,215 (97.74%)	ref	
Yes	2640 (2.26%)	1.40 (1.15, 1.72)	0.0009

**Fig. 1:**
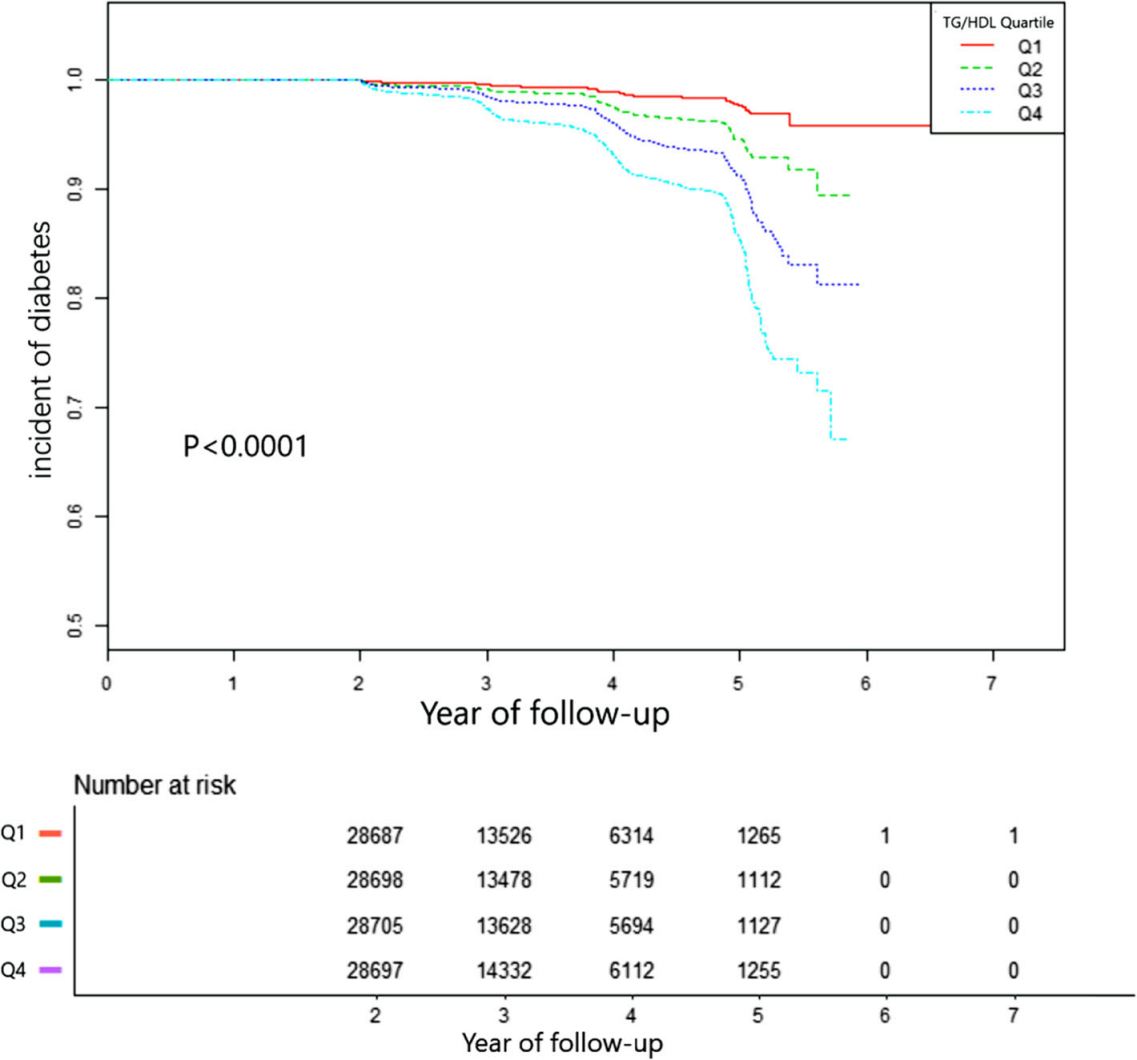
Kaplan–Meier event-free survival curve. Kaplan–Meier event-free survival curve. Kaplan–Meier analysis of incident of diabetes based on TG/HDL-C quartiles (logrank, *P* < 0.0001)

Figure 1 showed the Kaplan-Meier curves of the cumulative hazards of diabetes incident risk stratified by TG/HDL-C categories. Diabetes incident risk between each of the four TG/HDL-C groups was significantly different (log-rank test, *p* < 0.0001). With increased TG/HDL-C, the cumulative diabetes incident risk gradually increased, rendering the top quartile group with the maximum diabetes incident risk.

### The results of relationship between TG/HDL-C and incident of diabetes

We used cox proportional hazard regression model to evaluate the associations between TG/HDL-C and incident of diabetes. Meanwhile, we showed the non-adjusted and adjusted models in Table 3. In crude model, TG/HDL-C showed positive correlation with incident of diabetes (HR = 1.830, 95% confidence interval (CI):1.760 to 1.903, *P* < 0.00001). In minimally adjusted model (adjusted age, gender, BMI, SBP, DBP, family history of diabetes, smoking and drinking status), the result did not have obvious changes (HR: 1.301, 95% CI: 1.242–1.362). After adjusting for the full model (adjusted age, gender, BMI, SBP, DBP, FPG, TC, LDL, Scr, smoking and drinking status, family history of diabetes), we could also detect the connection (HR = 1.159, 95%CI: 1.104 to 1.215,*P* < 0.00001). For the purpose of sensitivity analysis, we also handled TG/HDL-C as categorical variable (Quartile), the top quartile had 70% increment of diabetes risk when compared with the bottom quartile in the full model, and found that the trend across the quartiles was significant (P for trend< 0.00001).

**Table 3:** Relationship between TG/HDL-C and the incident of diabetes in different models

Variable	Crude model (HR,95% CI,P)	Model I (HR,95% CI,P)	Model II (HR,95% CI,P)
TG/HDL-C	1.830 (1.760, 1.903) < 0.00001	1.301 (1.242, 1.362) < 0.00001	1.159 (1.104, 1.215) < 0.00001
TG/HDL-C(quartile)			
Q1	Ref	Ref	Ref
Q2	2.248 (1.890, 2.674) < 0.00001	1.413 (1.186, 1.683) 0.00011	1.321 (1.108, 1.574) 0.00187
Q3	3.765 (3.201, 4.427) < 0.00001	1.664 (1.409, 1.966) < 0.00001	1.445 (1.221, 1.709) 0.00002
Q4	6.625 (5.681, 7.726) < 0.00001	2.252 (1.914, 2.649) < 0.00001	1.700 (1.443, 2.003) < 0.00001
P for trend	< 0.00001	< 0.00001	< 0.00001

Crude model:we did not adjust other covariants

Model I:we adjust age, gender, BMI, SBP, DBP, family history of diabetes, smoking and drinking status

Model II: we adjust age, gender, BMI, SBP, DBP, FPG, TC, LDL-C, Scr, smoking and drinking status, family history of diabetes

*CI* Confidence, *Ref* Reference

### The analyses of non-linear relationship

**Fig. 2:**
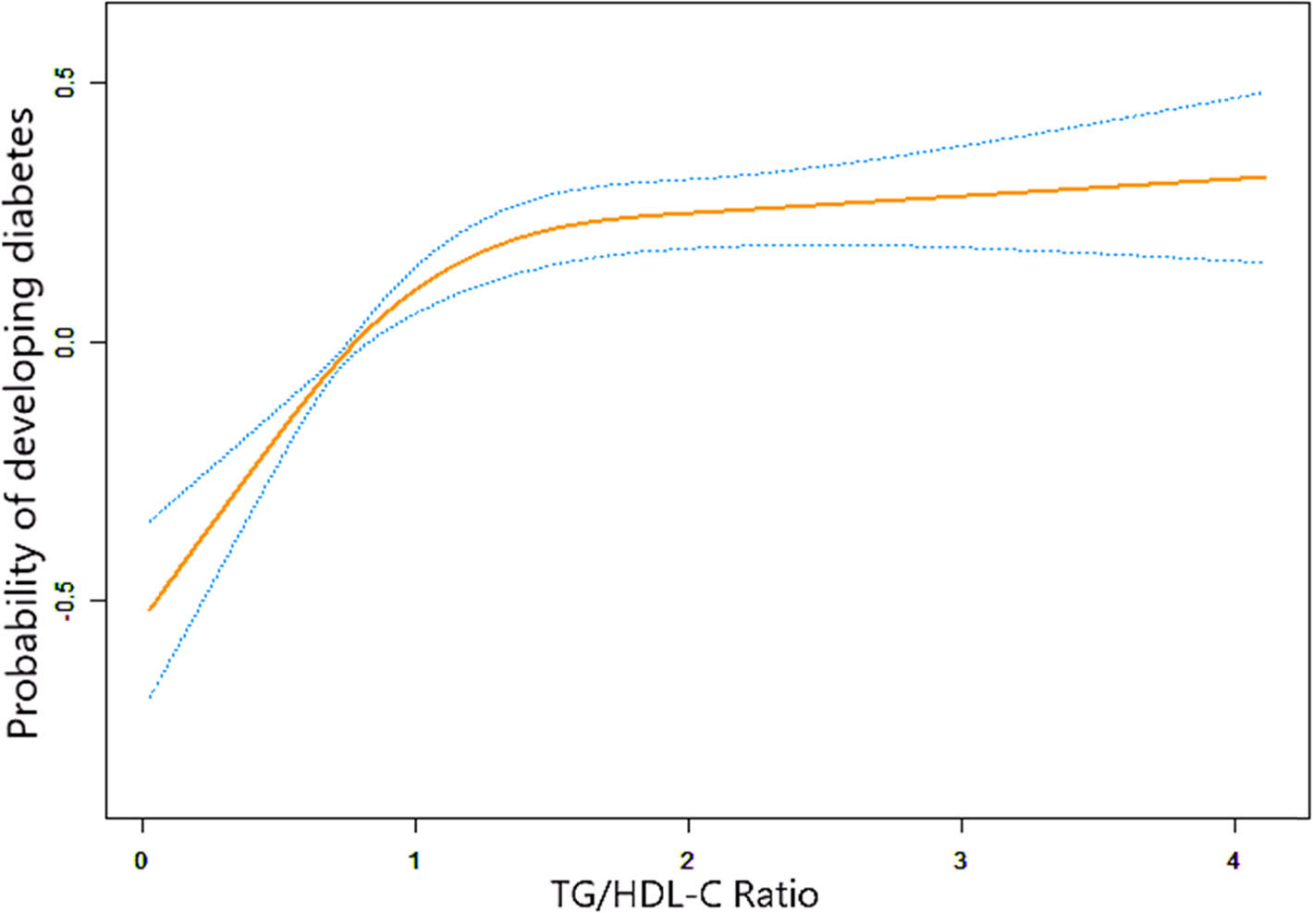
The non-linear relationship between TG/HDL-C and incident of diabetes. The non-linear relationship between TG/HDL-C and incident of diabetes. A non-linear relationship between them was detected after adjusting for age, gender, BMI, SBP, DBP, FPG, TC, LDL-C, Scr, family history of diabetes, smoking and drinking statuses

In the present study, we also used generalized additive model (GAM) to identify the non-linear relationship TG/HDL-C and incident of diabetes because TG/HDL-C was continuous variable (Fig. 2). We found that the relationship between TG/HDL-C and incident of diabetes was also non-linear (after adjusting age, gender, BMI, SBP, DBP, FPG, TC, LDL, Scr, smoking and drinking status and family history of diabetes). By using a two-piecewise linear regression model, we calculated that the inflection point of TG/HDL-C was 1.186 (Log- likelihood ratio test *P* < 0.001). On the left of the inflection point, we observed a positive relationship between TG/HDL-C and incident of diabetes(HR:1.718, 95% CI: 1.433–2.060,*P* < 0.0001). On the right side of the inflection point, however, their relationship tended to be saturated (HR: 1.049, 95% CI: 0.981–1.120, *P* = 0.060) (Table 4).

**Table 4:** The result of two-piecewise linear regression model

	Incident of diabetes	(HR,95%CI, P)
Fitting model by standard linear regression	1.159 (1.104, 1.215)	< 0.0001
Fitting model by two-piecewise linear regression		
Inflection point of TG/HDL-C	1.186	
≤ 1.186	1.718 (1.433, 2.060)	< 0.0001
> 1.186	1.049 (0.981, 1.120)	0.1600
P for log likelihood ratio test	< 0.001	

We adjusted age, gender, BMI, SBP, DBP, FPG, TC, LDL-C, SCR, family history of diabetes, smoking and drinking statuses

*CI* Confidence interval

### The results of subgroup analyses

We further explored other risks in the associations between TG/HDL-C and incident of diabetes by performing a subgroup analysis to estimate the factors that might influence the results, We used age, gender, FPG, BMI, SBP, DBP, family history of diabetes, smoking and drinking status as the stratification variables to observe the trend of effect sizes in these variables (Table 5). We noted that only a small number of interactions were observed based on our a priori specification including: FPG, BMI and family history of diabetes (all *P* values for interaction < 0.05). In this study, the stronger association were detected in the population with FPG < 6.1 mmol/L, BMI (≥18.5, < 24 kg/m^2^) and family history without diabetes. In contrast, the weaker association were detected in the population with FPG ≥ 6.1 mmol/L, BMI (< 18.5 or ≥ 24 kg/m^2^) and family history with diabetes.

**Table 5:** Effect size of TG/HDL-C on diabetes in prespecified and exploratory subgroups

Characteristic	No of participants	HR (95%CI)	*P* value	P for interacion
Age(years)				0.3871
20 to < 30	11,204	1.276 (0.745, 2.187)	0.3749	
30 to < 40	41,985	1.147 (0.974, 1.352)	0.1009	
40 to < 50	27,171	1.240 (1.108, 1.388)	0.0002	
50 to < 60	19,569	1.089 (0.998, 1.190)	0.0561	
60 to < 70	12,024	1.067 (0.970, 1.174)	0.1850	
≥70	4902	1.067 (0.934, 1.218)	0.3396	
Gender				0.0584
Male	62,868	1.114 (1.054, 1.177)	0.0001	
Female	53,987	1.233 (1.126, 1.350)	< 0.0001	
FPG				< 0.0001
< 6.1	112,483	1.296 (1.213, 1.384)	< 0.0001	
≥ 6.1	4372	1.051 (0.981, 1.126)	0.1600	
BMI				0.0100
< 18.5	5991	0.745 (0.180, 3.083)	0.6842	
≥ 18.5, < 24	63,581	1.324 (1.206, 1.453)	< 0.0001	
≥ 24, < 28	37,151	1.143 (1.065, 1.225)	0.0002	
≥ 28	10,132	1.067 (0.974, 1.169)	0.1616	
SBP				0.0903
< 140	104,008	1.180 (1.113, 1.250)	< 0.0001	
≥ 140	12,847	1.080 (0.992, 1.176)	0.0768	
DBP				0.8997
< 90	106,671	1.151 (1.090, 1.215)	< 0.0001	
≥ 90	10,184	1.143 (1.034, 1.263)	0.0091	
Smoking status				0.6833
Never smoker	24,684	1.154 (1.004, 1.327)	0.0441	
Ever smoker	1328	0.869 (0.540, 1.398)	0.5620	
Current smoker	6672	1.173 (1.007, 1.366)	0.0406	
Not recorded	84,169	1.160 (1.098, 1.225)	< 0.0001	
Drinking status				0.1344
Never drinker	878	1.175 (1.050, 1.314)	0.0048	
Ever drinker	5535	0.908 (0.708, 1.165)	0.4475	
Current drinker	26,273	1.554 (1.010, 2.391)	0.0450	
Not recorded	84,169	1.159 (1.097, 1.224)	< 0.0001	
Family history of diabetes				0.0251
No	114,215	1.170 (1.114, 1.228)	< 0.0001	
Yes	2640	0.856 (0.649, 1.129)	0.2719	

Note 1:Above model adjusted for age,gender,BMI,SBP,DBP,FPG,TC,LDL-C,SCR,smoking and drinking status,family history of diabetes

Note 2:In each case, the model is not adjusted for the stratification variable

### The results of relationship between other lipid parameters and incident of diabetes

After adjusting for the full model, we analyzed the associations between TG, HDL-C, TG/LDL-C, LDL-C/HDL-C, TC/HDL-C, TC/LDL-C and DM risk. We detected TG (HR = 1.414, 95% CI:1.414 to 1.573), HDL-C (HR = 2.807, 95% CI: 2.367 to 3.329), TG/LDL-C (HR = 1.765, 95% CI: 1.586 to 1.964) were positively connected with DM risk, moreover, we also found that LDL-C/HDL-C (HR = 0.818, 95%CI: 0.765 to 0.874), TC/HDL-C (HR = 0.738, 95% CI: 0.699 to 0.779), TC/LDL-C (HR = 0.652, 95%CI: 0.525 to 0.810) were negatively correlated with DM risk (Table S1).

## Discussion

Our findings indicated TG/HDL-C was positively associated with incident of diabetes after adjusting other covariates. Besides, we also found trend of the effect sizes on the left and right sides of the inflection point was not consistent [left (HR: 1.718, 95%CI: 1.433–2.060, *P* < 0.0001);right (HR: 1.049, 95%CI: 0.981–1.120, *P* = 0.060)]. This result suggested a saturation effect on the independent association between TG/HDL-C and incident of diabetes. Subgroup analysis will help us to better understand the trend of TG/HDL-C and incident of diabetes in different populations. The results of this study found the stronger association were detected in the population with FPG < 6.1 mmol/L, BMI (≥18.5, < 24 kg/m^2^) and family history without diabetes. In contrast, the weaker association between TG/HDL-C and incident of diabetes were detected in the population with FPG ≥ 6.1 mmol/L, BMI (< 18.5 or ≥ 24 kg/m^2^) and family history of diabetes.

Central obesity, insulin resistance, dyslipidaemia, and hypertension increase the risk of cardiovascular diseases (CVD) and DM. Triglycerides and HDL-C are important risk factors for cardiovascular disease [17]. As reported, although hypertriglyceridaemia might be associated with increased risk of CVD, the association is weakened when adjustment is made for other risk factors, particularly HDL-C levels, which often accompany elevated plasma triglyceride levels. However, even after adjustment for HDL-C levels, elevated triglyceride levels remain a risk factor for CVD [18]. Thus, TG/HDL-C has been proposed as a more practical and to easy use atherogenic marker, some researchers found it was a good marker for CVD [19–21]. Such metabolic perturbations are frequently associated with insulin resistance, and commonly associated with diabetes and metabolic syndrome. In our research, we found TG and HDL-C were risk factor for DM in Table S1, which was consistent with previous similar researches originated from lin et al. [5].

In early years, TG/HDL-C has been put forward to assess insulin resistance [22]. In recent years, researches have elucidated the correlations between TG/HDL-C and incident of diabetes. Some previous prospective studies have shown that high TG/HDL-C increase the risk of developing diabetes mellitus, such as Koreans [23] and Americans [10]. In Chinese population, He et al. [7] found in a retrospective study of 687 adults in an urban community located in Chengdu, Sichuan province, China that TG/HDL-C was one of independent DM risk factors and they observed an increasing trend of T2DM risk with TG/HDL-C after adjusting for potential confounders. Another study [9] enrolled 10,741 rural Chinese has similar finding. Consistently the same result that, we obtained cox proportional hazard regression model showed a significant and strong association between TG/HDL-C and incident of diabetes. In comparison, our research had a larger sample (114787) and from 32 sites and 11 cities in China, which was more representative of the Chinese population. While Janghorbani et al. [11] reported TG/HDL-C was not robust predictors of type 2 diabetes in high-risk individuals in Iranian. In a separate article [24], the authors reported that the plasma logarithm of the triglyceride/HDL-cholesterol ratio is a predictor of low risk gestational diabetes in early pregnancy. We analyzed these studies that are inconsistent with our results, and we speculate that the reasons for the different results may be caused by the following factors: (1) the research population is different. These studies, which were inconsistent with our findings, were targeted at Iranian and Euro-Brazilian pregnant women. (2) these different conclusions do not clarify the nonlinear relationship, (3) compared with our work, the study did not take into account the effect of BMI, SBP, DBP, TC, LDL, Scr, smoking and drinking status, family history of diabetes, on the TG/HDL-C and incident of diabetes relationships when adjusting covariates. However, previous studies have confirmed that these variables are related to TG/HDL-C or incident of diabetes, (4) this may be related to different races or gender,some studies showed that the association of TG/HDL-C and insulin resistance differ between races or gender [25–27]. In short, our results further confirmed that TG/HDL-C was positively associated with diabetes risk in Chinese cohort.

In the present study, the result we found using two-piecewise linear regression model to show a nonlinear relation is similar to that obtained by Cheng et al. [9]. In their study, they used restricted cubic spline to assess a nonlinear relation between TG/HDL-C and risk of T2DM, they found inflection point was 2.5, but they did not mention potential confounders. In our study, however, the inflection point obtained from GAM after adjusting for potential confounders (age, gender, BMI, SBP, DBP, FPG, TC, LDL, Scr, family history of diabetes, smoking and drinking statuses) was 1.186. Therefore, their conclusions was limited because they didn’t control potential confounders. Our study showed that when TG/HDL-C is below 1.186, the risk of diabetes increases with increasing TG/HDL-C levels, these people should pay more attention to prevent the risk of diabetes. The findings of this study should be helpful for future research on the establishment of diagnostic or predictive models of the risk of diabetes.

Our study have some strengths. (1) Our sample size is relatively large compared with previous similar studies; (2) we addressed the nonlinearity in the present study and further explore this; (3) this study was an observational study and therefore susceptible to potential confounding. We used strict statistical adjustment to minimize residual confounders; (4) We handled target independent variable as both continuous variable and categorical variable. Such an approach can reduce the contingency in the data analysis and enhance the robustness of results; (5) the effect modifier factor analysis maked the use of data better and yield stable conclusion in different subgroups in this study.

Potential limitations should be noted. Firstly, the cohort was conducted by the Rich Healthcare Group in China, and the data has been screened by Chen el at [12]. Due to raw data limitations, we could not conclude that our findings are suitable for people in other areas of different race and some special groups, such as pregnant women, children. Similarly, this study is based on a secondary analysis of published data, so variables that are not included in the data set cannot be adjusted, such as hip circumference,very low density lipoprotein, Interleukin-6 (IL-6), tumor necrosis factor (TNF). We could further explore their relationship with diabetes risk through collecting our data in the future. Secondly, similar to some articles [7], diabetes was defined as fasting plasma glucose of ≥7.00 mmol/L and/or self-reported diabetes during the follow-up period, rather than 2-h oral glucose tolerance test or measurement of glycosylated hemoglobin level, which may underestimate the incidence of diabetes. However, oral glucose tolerance tests for all participants were not feasible for pragmatic reasons and logistics. Thirdly, we only measured TG/HDL-C at baseline, which changes over time are not concerned in this study. Finally, even though we adjusted for an extensive set of confounding factors, residual confounding due to the measurement error in the assessment of confounding factors, unmeasured factors such as physical activity and dietary factors cannot be excluded. Further investigations in a longer follow up with more meticulous method are needed.

## Conclusion

TG/HDL-C is positively associated with diabetes risk. The relationship between TG/HDL-C and incident of diabetes is also non-linear. TG/HDL-C is positively related with incident of diabetes when TG/HDL-C is less than 1.186. In addition, the stronger association of TG/HDL-C and diabetes incident are detected in the population with FPG < 6.1 mmol/L, BMI (≥18.5, < 24 kg/m^2^) and family history without diabetes.

## Supplementary information

**Additional file 1: Table S1.** Relationship between other lipid parameters and the incident of diabetes in different models.

## Data Availability

Data can be downloaded from ‘DATADRYAD’ database (www.Datadryad.org).

## Abbreviations

BMI: Body mass index
BUN: Serum urea nitrogen
CVD: Cardiovascular diseases
DBP: Diastolic blood pressure
DM: Diabetes mellitus
FPG: Fasting plasma glucose
GAM: Generalized additive models
HDL-C: High-density lipoprotein cholesterol
LDL-C: Low density lipoprotein cholesterol
SBP: Systolic blood pressure
Scr: Serum creatinine
T2DM: Type 2 diabetes
TC: Total cholesterol
TG: Triglyceride
TG/HDL-C: Triglyceride to high-density lipoprotein cholesterol ratio
