# Supplementary material for: Association of Triglyceride to high-density lipoprotein cholesterol ratio and incident of diabetes mellitus: a secondary retrospective analysis based on a Chinese cohort study
Source: Lipids Health Dis. 2020 Mar 4;19:33. doi: 10.1186/s12944-020-01213-x (PMC7057518; doi:10.1186/s12944-020-01213-x)
Supplement: Supplementary file 1 — Additional file 1: Table S1. Relationship between other lipid parameters and the incident of diabetes in different models. [file 12944_2020_1213_MOESM1_ESM.pdf]

Supplementary Appendix

Supplementary table referred to in main text

Table S1 Relationship between other lipid parameters and the incident of diabetes in different models

Variable	Crude model(HR,95%CI,P)	Model I (HR,95%CI,P)	Model II (HR,95%CI,P)
TG	1.926 (1.855, 2.000) <0.00001	1.381 (1.321, 1.444) <0.00001	1.492 (1.414, 1.573) <0.00001
HDL-C	0.559 (0.490, 0.638) <0.00001	1.100 (0.956, 1.266) 0.18162	2.807 (2.367, 3.329) <0.00001
TG/LDL-C	3.480 (3.212, 3.769) <0.00001	1.983 (1.797, 2.188) <0.00001	1.765 (1.586, 1.964) <0.00001
LDL-C/HDL-C	1.376 (1.313, 1.441) <0.00001	0.958 (0.907, 1.012) 0.12137	0.818 (0.765, 0.874) <0.00001
TC/HDL-C	1.297 (1.257, 1.337) <0.00001	0.972 (0.936, 1.009) 0.13538	0.738 (0.699, 0.779) <0.00001
TC/LDL-C	1.089 (0.930, 1.276) 0.29053	1.113 (0.967, 1.282) 0.13636	0.652 (0.525, 0.810) 0.00011

Crude model:we did not adjust other covariants

Model I:we adjust age,gender,BMI,SBP,DBP， family history of diabetes,smoking and drinking status

Model II: we adjust age,gender,BMI,SBP,DBP,FPG,TC,LDL,HDL,TG,CCR,smoking and drinking status,family history of diabetes,and in each case,the model is not adjusted for the variable

CI confidence,Ref reference
